# Current Status and Challenges and Future Trends of Deep Learning-Based Intrusion Detection Models

**DOI:** 10.3390/jimaging10100254

**Published:** 2024-10-14

**Authors:** Yuqiang Wu, Bailin Zou, Yifei Cao

**Affiliations:** 1 College of Information and Technology, Nanjing Police University, Nanjing 210023, China; 114109@nfpc.edu.cn; 2 College of Artificial Intelligence, Nanjing Agricultural University, Nanjing 210095, China; 3 School of Electrical Engineering, Anhui Polytechnic University, Wuhu 241000, China

**Keywords:** deep learning, intrusion detection models, cybersecurity research, data preprocessing techniques, BERT and GPT models, future research directions

## Abstract

With the advancement of deep learning (DL) technology, DL-based intrusion detection models have emerged as a focal point of research within the domain of cybersecurity. This paper provides an overview of the datasets frequently utilized in the research. This article presents an overview of the widely utilized datasets in the research, establishing a basis for future investigation and analysis. The text subsequently summarizes the prevalent data preprocessing methods and feature engineering techniques utilized in intrusion detection. Following this, it provides a review of seven deep learning-based intrusion detection models, namely, deep autoencoders, deep belief networks, deep neural networks, convolutional neural networks, recurrent neural networks, generative adversarial networks, and transformers. Each model is examined from various dimensions, highlighting their unique architectures and applications within the context of cybersecurity. Furthermore, this paper broadens its scope to include intrusion detection techniques facilitated by the following two large-scale predictive models: the BERT series and the GPT series. These models, leveraging the power of transformers and attention mechanisms, have demonstrated remarkable capabilities in understanding and processing sequential data. In light of these findings, this paper concludes with a prospective outlook on future research directions. Four key areas have been identified for further research. By addressing these issues and advancing research in the aforementioned areas, this paper envisions a future in which DL-based intrusion detection systems are not only more accurate and efficient but also better aligned with the dynamic and evolving landscape of cybersecurity threats.

## 1. Introduction

**Figure 1:**
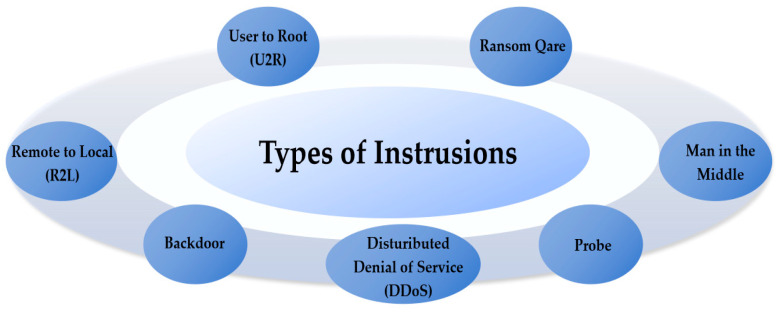
Common types of network intrusions.

The rapid advancement of the Internet has made networks essential tools for both personal and professional use. Simultaneously, zero-day vulnerabilities [1,2], mining Trojans, worms, and other forms of attacks have become increasingly prevalent in the network environment. Figure 1 illustrates common types of network intrusions. For a long time, firewall, policy management, encryption, authentication, and other preventive security mechanisms served as the primary line of defense against network intrusion [3]. However, these mechanisms are passive protection strategies. The security level they provide is insufficient to defend against internal attacks, and they rely heavily on historical traffic data. As a result, intrusion detection technology that actively addresses network anomalies has emerged.

Proactive intrusion detection techniques for addressing network anomalies focus on monitoring network traffic and activities within cybersecurity systems to actively identify and counter potential security threats. The essence of these technologies lies in moving beyond post hoc analysis and response to detecting anomalous behaviors and traffic in the network, swiftly identifying signs of potential attacks, and implementing appropriate defensive measures before threats materialize. In contrast to traditional passive intrusion detection systems, proactive intrusion detection techniques are more anticipatory and preventive. They can handle a diverse range of threat scenarios and are particularly effective at dealing with unknown attacks and zero-day exploits. These technologies typically integrate various detection methods, such as machine learning algorithms and big data analytics. By actively monitoring and analyzing network activities, the system can promptly intercept or alert administrators about potential threats before attack behaviors cause substantial harm, thereby minimizing security risks to the greatest extent possible. The application of such technologies has transitioned network security from a reactive to a proactive defense stance, thereby offering more effective strategies to counter cyber threats.

Generally, intrusion detection systems (IDSs) can be categorized into two types based on their data sources and detection technologies. According to their data sources, IDSs can be divided into host-based IDSs (HIDSs) and network-based IDSs (NIDSs) [4]. Based on different detection technologies, they can be divided into misuse-based IDSs (MIDSs) and anomaly-based IDSs (AIDSs) [5,6]. MIDSs evaluate network traffic against a database of known signatures; upon detecting a match, they activate an alert to indicate suspicious activity. However, MIDSs can only detect known attacks and cannot identify new threats. In contrast, AIDSs have the capability to identify unknown attacks and emerging security threats, thereby addressing this limitation. As a result, AIDSs have garnered significant attention from researchers.

AIDSs typically model behavior within the network to identify specific characteristics. AIDSs can make prior judgments and issue early warnings if they detect behavior that deviates from established norms during continuous network traffic monitoring. From the perspective of model learning, AIDSs include statistical analysis-based anomaly detection technology, traditional machine learning (ML), and deep learning (DL).

According to statistical analysis, AIDSs [7] do not rely on abnormal feature libraries and can detect unknown types of abnormalities. However, they are not appropriate for the current scenario of massive traffic data since they require the analysis of complex network data. Traditional ML detection techniques typically rely on the manual selection of features, which is considered shallow learning. The problem should be broken down into several parts before solving each part one by one and then devising a solution without taking into account the space-time characteristics of network traffic. However, increased network bandwidth results in an increase in massive network data, which, in turn, increases data complexity and diversity of features. This makes it challenging to accomplish analysis and detection goals with shallow learning. DL-based intrusion detection [8,9,10] integrates feature extractors and classifiers into a framework, which can automatically learn effective features and directly complete end-to-end training without using manually designed features.

Therefore, this article analyzes intrusion detection technology and related models based on DL, focusing on the references related to the topic of DL intrusion detection published and cited over the past seven years (from 2018 to 2024). It provides scholars with a comprehensive understanding of the current research trends. This article also reviews newly developed intrusion detection technologies, highlighting their advantages and disadvantages. Finally, it summarizes the latest trends in DL-based intrusion detection, emphasizes various challenges, and offers different future directions in this important field to promote the development of IDSs.

## 2. Dataset of Network Intrusion Detection

Selecting a detection dataset is a crucial step in intrusion detection research. There are typically two approaches to obtaining network traffic data: direct collection or using the public dataset. Direct collection involves using various software tools to capture network data packets. This method is advantageous for its specificity and is suitable for collecting small data. Conversely, with an extensive volume of data, the duration of gathering and the expenses for data retention are anticipated to rise. To save data collection time and improve efficiency, researchers typically choose existing public datasets. In the forthcoming section, we will highlight several online datasets [11]. After reviewing the literature on network intrusion detection over the past five years, we have identified seven of the most widely used datasets. The details are as follows.

### 2.1. KDDCup99

The KDDCUP99 [12] dataset includes 9-week network traffic from a simulated US Air Force LAN. The researchers used 7-week network traffic as an identified training set and the remaining 2-week network traffic as an unidentified test set. Attacks were categorized into the following four types: Probe, DOS, U2R, and R2L. Although the KDDCup99 dataset is relatively large, encompassing 41 distinct variables and surpassing 4.8 million records, it is subject to duplication between training and test data [13]. Furthermore, since the data were collected over 20 years ago, they have become outdated compared to more recent research.

### 2.2. NSL-KDD

The KDDCUP99 dataset is replete with numerous duplicate records, causing bias in the learning algorithm. To address this issue, related organizations have revised and cleaned the dataset. NSL-KDD [14] is an updated version of KDDCUP99, which is widely used for anomaly detection. NSL-KDD is composed of the following four subsets: KDDTrain+ and KDDTrain_20% for training purposes and KDDTest+ and KDDTest-21 for testing.

### 2.3. UNSW-NB15

The dataset known as UNSW-NB15 [15] was established in 2015. It contains nine different types of attacks. It also includes 49 features and about 2 million records. The dataset was assembled by employing Bro-IDSs and a number of novel algorithms.

### 2.4. CIC-IDS 2017

The CIC-IDS2017 dataset [16] was created over a span of five days, utilizing authentic devices to generate dynamic, real-world network traffic. One of the features of the CIC-IDS2017 dataset is that it includes a large volume of traffic data, encompassing both normal traffic and a variety of attack types. This dataset is extensively used for research in the field of network intrusion detection using machine learning and deep learning methods. This dataset reflects the complex and real network traffic of today’s infrastructure. The CIC-IDS2017 dataset encompasses a variety of frequently occurring attacks, including SSH brute force, Heartbleed, botnet activities, DoS, and DDoS attacks, etc.

### 2.5. Kyoto2006+

The Kyoto2006+ dataset [17] comprises network traffic data collected by Kyoto University through the use of honeypots, email servers, web crawlers, and additional network security mechanisms. Spanning from 2006 to 2015, this dataset encompasses 24 attributes, 14 of which are derived from the KDDCUP99 dataset, with an additional 10 attributes included. Each record is labeled as either normal (1), attack (−1), or unknown attack (−2).

### 2.6. ISCX2012

The ISCX2012 dataset [18] comprises a seven-day collection of pristine network traffic datasets, which encompasses both legitimate traffic flows and a quartet of adversarial traffic streams, namely, brute force SSH incursions, DDoS, HttpDoS, and infiltration activities. Compared to other datasets, the advantage of this dataset is that its attack types are modern and more realistic.

### 2.7. MQTTset2020

The MQTTet dataset [19], provided by Kaggle, originally consisted of 60 features. The data were collected from 10 types of devices, including thermometers, motion sensors, humidity sensors, etc. This dataset includes both normal data, as well as data for the following five types of attacks: DoS, brute force, malformed, SlowITe, and flood.

### 2.8. Brief Summary

In summary, the seven datasets discussed in this paper each offer unique perspectives and challenges for IDS research. The KDDCup99, despite its age and issues with data duplication, remains a foundational dataset in the field. NSL-KDD addresses the redundancy in KDDCup99 by offering a refined version for anomaly detection. UNSW-NB15 and CIC-IDS2017 offer more contemporary data, with CIC-IDS2017 simulating real-world network traffic over a short period. Kyoto2006+ extends the time frame of data collection, offering a longitudinal view of network traffic, whereas ISCX2012 is notable for its modern attack types. Lastly, MQTTset2020 introduces Internet of Things (IoT) device data, reflecting the evolving landscape of network security. Of course, in addition to the seven datasets mentioned above, other datasets, such as the CIC IoT Dataset 2023 [20], UNSW-NB15 [21], and TON_SoT [22], are widely used in the field of IoT. Table 1 summarizes the abovementioned seven datasets from their year of creation, numbers of network attacks, and attack types.

## 3. Data Preprocessing Methods and Feature Engineering Techniques

Data preprocessing and feature engineering techniques are indispensable, as they directly influence the performance and accuracy of the detection system. Through meticulous data preprocessing and feature engineering, network intrusion detection systems can more accurately identify and differentiate between normal and malicious traffic. This enhancement improves precision and response speed, which is crucial for constructing an effective IDSs. The intrusion detection model serves as a classifier capable of distinguishing normal data from abnormal within a dataset. Data preprocessing refers to a series of operations performed prior to primary tasks, such as model input. Feature engineering techniques are crucial for enhancing model performance, as they enable the model to effectively capture potential patterns and relationships within the data. While data preprocessing focuses on ensuring data quality, feature engineering typically aims to enhance model performance. The following sections introduce several commonly used data preprocessing methods and feature engineering techniques for intrusion detection models.

### 3.1. Common Data Preprocessing Methods

#### 3.1.1. Data Numerical Processing

Consider KDDCUP 99 as an example. This dataset contains character-type features, whereas DL-based intrusion detection models can only process numerical-type features [23]. To convert the character-type features into numerical-type values, two coding methods are typically used, namely, label coding and one hot coding [24]. The application of one hot encoding is a prevalent practice. Its fundamental principle involves encoding multiple states using multi-bit status registers. Initially, the technique of one hot encoding is implemented to transform categorical feature representations into a numerical format. This methodology bifurcates the dataset’s features into continuous and categorical dimensions. Within the KDDCUP99 dataset, the feature ‘protocol_type’ encompasses the following three discrete values: TCP, UDP, and ICMP. After applying one hot coding, the values of these three protocols are represented as [1, 0, 0], [0, 1, 0], and [0, 0, 1].

Consequently, this method may lead to the augmentation of the divergence among the distinct features, thus necessitating the standardization of the dataset’s feature values within the range [−1, 1]. The purpose is to compress the data without changing the original information. However, intrusion detection models based on DL typically require two-dimensional graphic data, while the KDDCUP99 is made up of one-dimensional data. Hence, it is imperative, during the data preprocessing phase, to transform the unidimensional data into bidimensional graphical representations.

#### 3.1.2. Data Standardization Processing

There are still significant differences in the values of each feature, even after the numerical processing of intrusion detection data. Without standardization, the gradient will disappear in the backpropagation algorithm, which can impede the growth of the learning weight and the threshold of the intrusion detection model, making it difficult to extract features effectively. Therefore, standardizing the intrusion detection data during the preprocessing stage is essential. For the model using gradient descent optimization, this standardization can significantly improve convergence speed. Widely recognized approaches for data standardization are the min–max standardization and the z-score standardization.

Min–max standardization is a linear transformation technique that projects the data onto a continuum scaled from 0 to 1 [25]. For instance, Liu et al. [26] used min–max standardization to preprocess the KDD999 and UNSW-NB15 datasets in preparation for subsequent model training and testing. Whereas z-score standardization entails the computation of the original dataset’s mean and standard deviation [27], thereby standardizing the data. This process transforms the dataset into a form that adheres to a standard normal distribution, characterized by a mean of 0 and a standard deviation of 1. To effectively apply z-score standardization, the original data should closely follow a Gaussian distribution; otherwise, it may impair model training. The z-score standardization method compresses the data to [−1,1].

#### 3.1.3. Handling Imbalanced Datasets

Model deficiencies reveal that certain IDS proposals exhibit reduced detection accuracy for specific attacks relative to the model’s aggregate rate, attributable to dataset imbalance. Low-frequency attacks have a lower detection accuracy than attacks with a higher number of instances. This issue can be addressed by improving techniques for handling low-frequency attack samples. Specifically, the synthetic minority oversampling technique (SMOTE) [28,29] generates new samples through interpolation within the feature space, thereby increasing the quantity of low-frequency attack samples and effectively balancing the dataset. This approach facilitates the model’s enhanced capability to discern the nuances of infrequent attack patterns. Furthermore, the application of generative models, such as generative adversarial networks (GANs) or variational autoencoders (VAEs), can be employed to generate realistic low-frequency attack samples. The amalgamation of these synthetic samples with the existing dataset is essential for constructing a balanced training dataset. This integration permits the re-education of the intrusion detection model and the subsequent assessment of its efficacy, with a spotlight on the detection rate of less common attacks. Such a methodology augments the dataset’s diversity and its capacity to represent a broad spectrum of attack vectors.

During model training, the strategic allocation of increased weights to samples representative of less frequent attack types compels the model to focus more intently on these instances, thus enhancing the detection rate for such attacks, all without necessitating any modification to the original dataset. Furthermore, combining oversampling and undersampling techniques can also optimize the sample distribution within the dataset. Oversampling increases low-frequency attack samples, while undersampling decreases high-frequency samples, thereby balancing the dataset to effectively detect all types of attacks. For instance, in their study on intrusion detection within industrial control systems, Ali et al. [30] addressed the challenge of multi-class imbalanced classification by employing a range of data preparation techniques. These techniques included normalization, Fisher’s discriminant analysis, and the KNN method, which were used for scaling, dimensionality reduction, and resampling of the data. Their approach culminated in achieving an accuracy rate of 99% on an industrial network dataset. As the network environment evolves and new attack types emerge, datasets should be regularly updated to include the latest attack patterns. Researchers can continuously expand and update the dataset to more accurately reflect current cybersecurity threats by collecting more low-frequency attack samples or employing generative AI technologies.

#### 3.1.4. Graphical Data Processing

Commonly used intrusion detection models necessitate that the input data be two-dimensional graphics. For instance, after Lin et al. [23] processed the data, its dimension was initially 122. After deleting the marked feature column, the dimension was reduced to 121. The one-dimensional data was then reshaped into an 11 × 11 matrix to serve as the model’s input. However, the data samples contained many 0 values, which impaired the CNN’s ability to perform an effective calculation. Therefore, the 0 values were regarded as gray values in the two-dimensional graph and ignored in the feature extraction process, facilitating the successive refinement of intricate feature sets from the initial low-level data inputs.

### 3.2. Common Feature Engineering Techniques

#### 3.2.1. Feature Selection and Dimensionality Reduction

L1 regularization, L2 regularization, and principal component analysis (PCA) are prevalent techniques employed for the purposes of feature subset selection and dimensionality reduction. By incorporating regularization terms (L1 or L2) into the loss function, feature weights can be restricted, facilitating effective feature selection. L1 regularization (Lasso) causes some feature weights to become zero, thus identifying the most significant features, whereas L2 regularization (Ridge) prevents model overfitting. Regularization techniques enable researchers to automatically identify the most crucial features during model training. PCA is a common dimensionality reduction method that minimizes data redundancy by converting the high-dimensional data into a lower-dimensional set of principal components. PCA is extensively utilized in intrusion detection for data dimensionality reduction to decrease computational complexity and enhance model efficiency.

#### 3.2.2. Deep Feature Extraction

Autoencoders and CNNs are common deep feature extraction techniques. Autoencoders are a learning technique that creates a low-dimensional representation of data through an encoder–decoder structure, making them useful for feature extraction. In intrusion detection, autoencoders can extract meaningful features from complex network data, enhancing the models’ ability to detect anomalous behavior. CNNs can be used not only for image recognition but also for extracting spatially correlated features by analyzing network traffic data in two dimensions. In intrusion detection, the transformation of unidimensional network traffic data into bidimensional imagery followed by the application of CNNs for feature extraction can markedly augment the model’s capacity to discern intricate attack signatures.

#### 3.2.3. Feature Construction

Feature construction often employs aggregation and interaction feature techniques. Aggregating certain features in network traffic data (such as the total traffic volume or number of requests within a given time frame) can lead to the creation of more representative features, thereby improving the model’s detection abilities. Additionally, generating interactive terms between features allows models to capture intricate relationships among them. For example, the synthesis of a novel feature through the amalgamation of the source IP address with the destination port number can significantly bolster the model’s efficacy in detecting specific categories of attacks.

## 4. Intrusion Detection Model Based on DL

Since Professor Hinton put forward the theory and technology of DL in 2006, several differences between DL and ML have been analyzed. Firstly, unlike traditional ML, which necessitates manual feature selection, DL can automatically learn effective features and perform end-to-end training directly. Secondly, DL is suited for handling large datasets. During the model training phase, DL requires more time compared to ML. However, during the testing phase, the advantages of DL algorithms become more apparent. Thirdly, DL can augment the process of feature learning by progressively refining feature representations through successive layers, thereby enhancing the precision of model predictions or classifications. Thus, the current research focuses on DL-based intrusion detection models.

In previous research, scholars have reviewed and summarized the DL-based intrusion detection models [31,32,33,34,35,36], but the coverage remains insufficient. This study presents seven types of intrusion detection models (IDMs), that is, IDMs based on deep autoencoders (DAE-IDMs), IDMs based on deep belief networks (DBN-IDMs), IDMs based on deep neural networks (DNN-IDMs), IDMs based on CNNs (CNN-IDMs), IDMs based on recurrent neural networks (RNN-IDMs), IDMs based on generative adversarial networks (GAN-IDMs), and IDMs based on transformers (TF-IDMs). Additionally, the application of large language models (LLM) in network intrusion detection is becoming more prevalent [37]. This paper also introduces the IDM based on the BERT series of models (BERT-IDMs), as well as those based on the GPT series of models (GPT-IDMs).

### 4.1. Introduction to DAE-IDMs

An autoencoder is an unsupervised learning algorithm used in deep learning designed to learn a compressed representation, referred to as an encoding, of the input data. Autoencoders constitute a significant instrument in the realm of intrusion detection, attributable to their proficiency in data learning and representation, anomaly detection, and data preprocessing for advanced analytical endeavors. Farahnakian et al. [33] used a deep autoencoder (DAE) to create an IDM. In comparison to traditional autoencoders, DAE increases the number of hidden layers. They trained DAE using the greedy unsupervised hierarchical training mechanism to prevent overfitting. The attack categories were classified using a soft-max classifier following the DAE’s training. The evaluation on KDDCUP99 demonstrated that DAE-IDMs achieved accuracies of 96.53% and 94.71% in binary classification (BC) and multi-classification (MC), respectively. However, the model evaluation index is relatively limited. Shone et al. [38] proposed a nonsymmetric DAE-IDS. The asymmetric deep AE does not contain a decoder and has multiple hidden layers. The nonsymmetric DAE-IDS was applied to both the KDDCUP99 and NSL-KDD using an RF classifier, achieving accuracies of 97.85% and 85.42%.

Khan et al. [39] proposed a novel two-stage semi-supervised feature learning model, TSDL. The architecture of the model was bifurcated into two distinct phases, each integrating a deep sparse autoencoder and a soft-max classifier. The model’s hidden layers were autonomously pre-trained on an extensive dataset of unlabeled network traffic features via an unsupervised methodology, followed by fine-tuning with labeled network traffic data. During the preliminary phase, the network traffic was bifurcated into normal or anomalous categories predicated on a probabilistic score. This probabilistic metric was subsequently leveraged as an ancillary feature for the model’s culminating stage, which entailed multi-class discrimination between normalcy and diverse attack vectors. Both the KDDCUP99 and UNSW-NB1 datasets demonstrated perfect accuracy and extremely low false positive rates. However, the calculation time increased as the dataset became larger.

A large volume of network traffic and high-dimensional features can complicate the classification process, making it tedious and complex. In addressing the challenge, Yan et al. [40] introduced a novel approach by utilizing a stacked sparse autoencoder (SSAE) to effectively extract sophisticated features. In comparison to the traditional autoencoder, the sparse autoencoder included a sparse penalty term in its hidden layer. The experiment achieved superior classification performance compared to other methods. It also exhibited optimal training time and required the fewest training samples. However, the model struggled to effectively detect low-frequency R2L and U2R attack samples, indicating that it could not address the issues caused by the unbalanced data distribution.

In studies [41,42], PCA or GFR was used for feature selection in SVM classifiers. However, both PCA and GFR are two feature selection technologies with high computational costs during training and testing. Additionally, with sparse autoencoders, increasing the number of hidden layers generally improves classifier performance but also extends the training time, potentially doubling it. To strike a balance between the classification performance and the training and testing duration, Al-Qatf et al. [43] introduced an integrated IDS, STL-IDS, which is constructed on the STL framework. This system integrates a sparse AE for the extraction of low-dimensional features, which are then passed to an SVM classifier for the classification process, eschewing the traditional use of a soft-max classifier. Evaluation with the NSL-KDD dataset indicated that STL-IDS achieves significantly reduced training and testing times compared to a standalone SVM classifier, particularly in scenarios involving two-class and five-class classifications. In addition to the recall rate, STL-IDS outperformed a single SVM classifier. Compared to other traditional ML methods, such as J48 and naive Bayes, STL-IDS achieved higher accuracy on NSL-KDD, particularly in the five categories. In addition to the two AE variants mentioned above, there is also a VAE. VA has a similar structure to that of automatic encoders as a whole. In work [44], VA was deployed for the purpose of intrusion detection. The findings from the CIC-IDS2017 dataset analysis revealed that the detection rate achieved by the VAE notably exceeded those of both AE and SVM.

### 4.2. Introduction to DBN-IDMs

The restricted Boltzmann machine (RBM), a class of stochastic neural networks, constitutes a binary-layered architecture devoid of differentiation between forward and inverse propagations, encompassing a discernible input stratum and an occult layer. Its proficiency in extracting salient high-level characteristics from intricate primary datasets renders it a favored instrument within the discourse of intrusion detection academia. Aldwairi et al. [45] proposed a framework that integrates an RBM with training algorithms such as contrastive divergence (CD) and persistent contrastive divergence (PCD). The model was rigorously trained, validated, and evaluated using a balanced dataset derived from the ISCX2012, showcasing its efficacy in intrusion detection tasks. The results indicated that RBM-IDSs effectively differentiated between normal and abnormal network behaviors and identified new attack models. The accuracy of the PCD algorithm was 89.7%, while that of the CD algorithm was 89.3%. The RBM constitutes a neural network architecture with a singular hidden layer. Upon augmentation of the hidden layers, the model progresses into a configuration known as the deep Boltzmann machine (DBM). In a DBM, nodes in all layers are undirected. However, if the connection relationship between some layers is restricted to be directed, it results in a different structure known as a deep belief network (DBN). DBNs can minimize dimensionality while preserving original features, making them suitable for classification.

Research has demonstrated that the application of DBNs for feature extraction, followed by the utilization of these features in an SVM classifier, yields superior classification performance [46]. The efficacy of this methodology was substantiated through rigorous testing on the KDDCUP99 dataset, in which it attained an accuracy level of 95.45%. This represents a significant enhancement of 11.58% over the traditional PCA approach and an improvement of 12.91% when compared to the gain ratio technique. Zhang et al. [47] also developed a DBN-SVM intrusion detection model. Experiments on the CIC-IDS2017 dataset demonstrated that this method achieved greater real-time detection efficiency compared to traditional ML algorithms. The accuracy of attack classification was 0.7% higher than that of the single DBN. Zhao et al. [48] integrated deep belief networks (DBN) with probabilistic neural networks (PNNs) for intrusion detection, employing particle swarm optimization (PSO) to optimize DBN’s hidden layer neurons. The KDDCUP99 dataset was used to test the above methods, and it was found that the overall performance of this method surpassed that of the PCA-PNN algorithm. This demonstrated that the dimension reduction effect of DBN was superior to that of PCA. However, this method’s false positive rate exceeded that of the individual PNN and the unoptimized DBN-PNN combination.

In another study [49], a hybrid approach was adopted for intrusion detection to curtail the training dataset and address network traffic imbalances. This approach integrated an enhanced density peak clustering algorithm (MDPCA) with DBN. The MDPCA segmented the extensive training data into smaller, more manageable subsets, each of which was used to independently train a sub-DBN classifier. These classifiers automatically condensed data dimensions post-feature extraction and achieved efficient classification. In the testing phase, outcomes from individual sub-DBNs were pooled based on fuzzy membership to determine the final output. Despite a higher false positive rate, this model demonstrated improved performance in accuracy, precision, and F1 score over conventional methods.

The elevated false positive rate suggests the model’s inconsistent accuracy across different attack types, as it performed well in some cases but not in others. Therefore, an adaptive model is urgently needed to handle diverse network structures. Zhang et al. [50] introduced an intrusion detection model leveraging GA to fine-tune the architecture of a DBN, optimizing layer depth and neuron count to enhance accuracy with a streamlined design. The experimental results, which used NSL-KDD, showed that the improved model could achieve a recall rate of more than 99%. At the same time, the classification accuracy for U2R attacks on a small training set reached 98.68%, which was also higher than that achieved by other algorithms.

The BP algorithm is commonly used to train neural networks; it trains models by randomly initializing weights and thresholds. However, this approach can lead to several issues. For instance, achieving a local optimal solution often requires a lengthy training cycle. Wang et al. [51] tackled this challenge by substituting the BP algorithm in DBN with a supervised kernel extreme learning machine (KELM). This approach aimed to enhance the generalization ability of a KELM classifier across various datasets. The KELM integrated the kernel function with ELM and used a nonlinear mapping method to project linearly non-separable information into a high-dimensional feature space, thereby achieving linear separability. In the DBN-EGWO-KELM model, Wang et al. [51] initially utilized DBN for feature dimension reduction. The processed dataset was then split into training, validation, and testing subsets for the EGWO-KELM classifier. They enhanced EGWO with a hybrid search strategy and applied it to optimize KELM’s parameters. Replacing the BP algorithm, the refined EGWO-KELM model was trained on the training and validation sets and evaluated on the test set. It showed high accuracy and stability across various datasets, like KDDCUP99, NSL-KDD, and CIC-IDS2017. However, it was less responsive to worm and backdoor attacks in the UNSW-NB15 dataset.

### 4.3. Introduction to DNN-IDMs

In the realm of deep learning, the deep neural network (DNN) represents one of the foundational neural network architectures, characterized by an extended hierarchy of layers beyond the standard input and output stratum, inclusive of multiple hidden layers. A pivotal challenge in the construction of DNNs pertains to the optimization of the hidden layer count and the neuron distribution within each layer. In pursuit of ascertaining the optimal configuration for DNN-IDMs, Vigneswaran et al. [52] systematically varied the number of hidden layers, ranging from 1 to 5, and executed a training regimen of 100 epochs utilizing the KDDCUP99 dataset. Their empirical analysis revealed that the DNN exhibited the most efficacious performance with an architecture comprising three hidden layers.

Ma et al. [53] introduced the SCDNN model, employing spectral clustering (SC) for data characterization and subset partitioning, followed by a DNN to discern and categorize subset features through iterative training and refinement. This approach enables the DNN to draw on prior knowledge, enhancing its understanding of diverse network attack paradigms. Evaluation against the KDD-CUP99 and NSL-KDD datasets confirmed SCDNN’s superior accuracy over SVM and RF, particularly for U2R and R2L classifications. However, DNN’s layer-specific weights and thresholds are determined empirically, lacking a theoretical underpinning.

Khare et al. [54] mitigated the challenge of high-dimensional network traffic in intrusion detection by deploying the spider monkey optimization (SMO) algorithm for dataset dimensionality reduction prior to DNN ingestion. The DNN model based on SMO achieved 99.4% and 92% accuracy, 99.5% and 92.7% accuracy, 99.5% and 92.8% recall, and 99.6% and 92.7% F1 values on the NSL-KDD and KDDCUP99 datasets, respectively. However, the model was only limited to the case of two classifications.

The imbalance of datasets has always been a challenge for researchers. At the same time, dimensionality reduction methods, such as PCA, mainly rely on artificial feature extraction, with their performance significantly influenced by luck and experience. To address these issues, Yang et al. [55] proposed a novel model that integrates the conditional variation automatic encoder (ICVAE) with a DNN, referred to as ICVAE-DNN. The model featured a trained decoder that synthesized new attack samples corresponding to predefined intrusion types. These samples were incorporated into the existing training data to equalize the dataset and broaden its variability. The ICVAE also served dual purposes of dimension reduction and initialization of the DNN’s hidden layers, streamlining the attainment of global optimization via backpropagation. Evaluation using the NSL-KDD and UNSW-NB15 datasets indicated that the ICVAE-DNN model surpassed ROS, SMOTE, and ADASYN in enhancing dataset diversity. It showed heightened efficacy in identifying specific (U2R, R2L) and novel attack vectors, including shellcode and worms. Nonetheless, there is room for improvement in terms of the model’s precision, sensitivity on the NSL-KDD dataset, and the false positive rate on the UNSW-NB15 dataset.

### 4.4. Introduction to CNN-IDMs

With the increase in network traffic, CNNs can learn more useful features to improve their classification capabilities. Hence, CNNs are apt for vast network environments and are widely applied in intrusion detection systems. Khan et al. [56] evaluated an augmented CNN model on a subset of the KDDCup99 dataset, optimizing kernel dimensions and pooling parameters for enhanced detection performance. Experiments revealed that the enhanced CNN model outperformed both SVM and DBN in detection, achieving an accuracy of 99.23%. However, the evaluation index of this experiment was single, without considering the false alarm rate, F1, etc. Riyaz et al. [57] introduced the CRF-LCFS algorithm for feature selection, followed by CNN classification, achieving 98.88% accuracy and a false positive rate below 1% on the KDDCup99 dataset, yet neglecting a comprehensive assessment including recall.

Wu et al. [58] used a variance algorithm to convert the original input into an image data format, which was then input into the convolution layer. Other experiments revealed that the poor detection performance of NSL-KDD for U2R attacks was due to the fact that “U2R” data represented only 0.04% of the dataset, whereas “normal” data comprised more than 50%. To address this issue, a cost function-based approach was implemented to assign weight coefficients to each class of training samples. The NSL-KDD dataset demonstrated that the proposed model surpassed conventional ML-based intrusion detection, realizing an average accuracy of 70.09% and a minimal false positive rate of 0.06% for “U2R” attacks, yet with significant potential for enhancement. To enhance IDS efficiency, increase the detection rate of minority classes, and address imbalances in large-scale datasets, Zhang et al. [59] introduced SGM, a novel technique that amalgamates SMOTE with a GMM-based undersampling strategy to devise an sSGM-CNN model. This model integrates class imbalance processing with CNN architecture. The study also analyzed the effects of kernel count and learning rate adjustments on model efficacy, achieving high detection rates on UNSW-NB15 and CIC-IDS2017 datasets.

In recent research, Wu et al. [60] integrated a CNN with a three-branch decision-making approach, called CNN-TWD. In this model, a CNN was employed for feature extraction, followed by a three-branch decision-making process for classifying network behavior. Based on the two-branch decision, the three branches decision introduced the concept of boundary domains. Generally, the method categorizes the network behavior into normal, intrusion, and undetermined. For the uncertain network behavior within the boundary domain, the three decision-making branches would pause the decision-making behavior and await additional feature extraction by the CNN to provide supplementary decision-making information before re-evaluating the network behavior. Experiments with the NSL-KDD dataset demonstrated that the CNN-TWD has higher accuracy, recall, and F1 than the PCA-TWD and DNN-TWD and has the best comprehensive performance. Another experiment revealed that the CNN-TWD algorithm continued to deliver the best overall performance, with an F1 that was 1% higher than the other best results. However, the threshold setting of three-branch decision-making needs further study.

Wang et al. [61] introduced HAST-IDS, which is an intrusion detection system leveraging hierarchical spatiotemporal features to tackle high false positive rates and the absence of time series analysis in IDSs. This model autonomously learns from raw network traffic data; it employs a CNN for spatial feature extraction at a basic level and short-term and long-term memory network (LSTM) for temporal feature abstraction at a higher level. Validated using the ISCX2012 dataset, HAST-IDS showed promising results, albeit at the cost of increased computational expenditure due to its dual-phase feature learning approach.

### 4.5. Introduction to RNN-IDMs

A key characteristic of an RNN is its capacity to maintain a loop of information through hidden layers, preserving processed data, which confers a structural advantage for managing time series data. Consequently, diverse intrusive activities can be conceptualized as distinct temporal sequences within the network fabric. This trait renders RNNs well-suited for the development of IDSs.

Yin et al. [62] proposed the RNN-IDS. During their experiments, they transformed 41 features from the NSL-KDD dataset into 122 features by mapping strings to binary. Their results demonstrated that the RNN-IDS achieved remarkable performance, with an accuracy rate of 83.28%. In their analysis of the RNN-IDS, they observed that both the quantity and learning rate of the RNN’s hidden nodes significantly impacted the system’s efficacy. Optimal performance was attained with a configuration of 80 hidden nodes at a learning rate of 0.5. Nonetheless, the evaluation was primarily constrained to accuracy. It is important to note that this RNN operates unidirectionally, meaning that each output is contingent upon the previous timestep’s input, without the benefit of future timestep data.

The aforementioned RNN is of the unidirectional variety, in which each output is contingent upon the preceding time step’s input, precluding the utilization of subsequent temporal data. Therefore, Shuster et al. [63] developed a bidirectional cyclic neural network (BRNN). The core concept of the BRNN is to process data through two separate hidden layers in both forward and backward directions while sharing the same input and output layers. The remaining components are similar to those of a standard RNN.

#### 4.5.1. IDM Based on an LSTM

RNNs are inherently equipped to handle sequences of limited duration; however, extensive sequences may encounter limitations due to short-term memory constraints. To mitigate these, advanced RNN forms like LSTM and GRU have been developed. LSTM networks, which are an enhancement of standard RNNs, are specifically crafted to tackle the challenges of vanishing and exploding gradients. They enable the capture of long-term dependencies through the utilization of input, forget, and output gates.

Su et al. [64] introduced the BAT-MC model, which comprises an input layer, multiple convolutional layers, a bidirectional LSTM (BLSTM) layer, an attention layer, and an output layer. This architecture translates numerical datasets into flow imagery for feature extraction, employing a convolutional approach. The BLSTM layer processes traffic data bidirectionally to capture temporal features. The attention mechanism evaluates the significance of features, highlighting those crucial for identifying malicious traffic. The output layer then consolidates these features, which are subsequently classified using a soft-max classifier. The BAT-MC model demonstrated enhanced classification accuracy over traditional methods on the NSL-KDD dataset, yet it underperformed in detecting U2R attacks and had a high false positive rate for normal traffic. Mirza et al. [65] presented an LSTM-based framework for automated sequential encoding and feature extraction, setting a threshold for anomaly detection based on cross-validation. This framework achieved an F1 score of 85.83% on the ISCX2012 dataset, suggesting the necessity for further refinements.

#### 4.5.2. IDM Based on a Gated Recurrent Neural Network (GRNN)

The gated recurrent unit (GRU) represents an advancement over the LSTM model, streamlined by merging the input and forget gates into a single update gate. This simplification reduces the computational and memory overhead compared to LSTM. Typically, GRUs conclude with a soft-max layer for classification. In [66], an SVM with a margin function replaced the soft-max layer in the GRU, achieving superior results over the Kyoto2006+ dataset, with training and testing accuracies of 81.54% and 84.15%, respectively, compared to the GRU soft-max model’s 63.07% and 70.75%. Xu et al. [67] introduced a time-aware IDS integrating a GRU, MLP, and soft-max module. The GRU was tasked with feature extraction and retention, the MLP provided nonlinear mapping and classification, and the soft-max layer normalized probabilities for the final output. While effective on KDDCUP99 and NSL-KDD, its detection of R2L and U2R attacks was suboptimal.

Vinayakumar et al. [68] capitalized on the temporal nature of network traffic by integrating a CNN with an RNN, LSTM, and GRU for intrusion detection. Their evaluation of the KDDCUP99 dataset revealed that a two-layer CNN-RNN model offered the highest accuracy, potentially reaching 100%. However, the complexity led to prolonged training times and overfitting, particularly for frequent attack types. An enhanced CNN-LSTM model [69] addressed overfitting with two convolutional layers, two pooling layers, LSTM, and three fully connected layers. The CNN functioned as a feature extractor, with dropout layers reducing overfitting, and LSTM-learned temporal features. Data were fed into fully connected layers with a dropout probability before classification via a soft-max classifier. Tuning the number of iterations and dropout probability optimized performance, with 100 iterations and a 0.2 dropout probability yielding the highest accuracy on the KDDCUP99 dataset.

### 4.6. Introduction to GAN-IDMs

GANs have emerged as a leading unsupervised learning approach, harnessing adversarial training between generators and discriminators to produce high-fidelity outputs. Given the scarcity of anomalous data in cybersecurity, GANs mitigate class imbalances by generating synthetic yet diverse attack samples from a modest dataset, thereby enhancing detection accuracy in IDS models, particularly when datasets are limited. Salem et al. [70] addressed the challenges associated with imbalanced datasets by generating synthetic anomalies. They initially converted the data into image format, then employed a cyclic Generative Adversarial Network (GAN) to create images of anomalies from images of normal data. The synthesized data was subsequently integrated with the original dataset for training models to detect anomalies. This methodology resulted in improved classification outcomes, with the area under the Receiver Operating Characteristic (ROC) curve increasing from 0.55 to 0.71, and the anomaly detection rate escalating from 17.07% to 80.49%. Moreover, a comparative analysis with SMOTE was performed, highlighting the potential of GANs in the generation of anomalies.

To counteract the skewed detection rates due to imbalanced traffic, Li et al. [71] introduced a GAN-based IDS model. This model employed information gain and PCA for feature extraction, followed by DBSCAN for data clustering and generation. Experiments across three datasets using six classifiers, including XGBoost, achieved accuracies ranging from 90% to 98%.

In addition, Liu et al. [72] proposed a GAN-based strategy to address the imbalance and high dimensionality in intrusion detection datasets. GANs were used to augment minority class samples, while variance analysis was applied for feature selection, resulting in a balanced, low-dimensional dataset for ML training. 

### 4.7. Introduction to TF-IDMs

**Figure 2:**
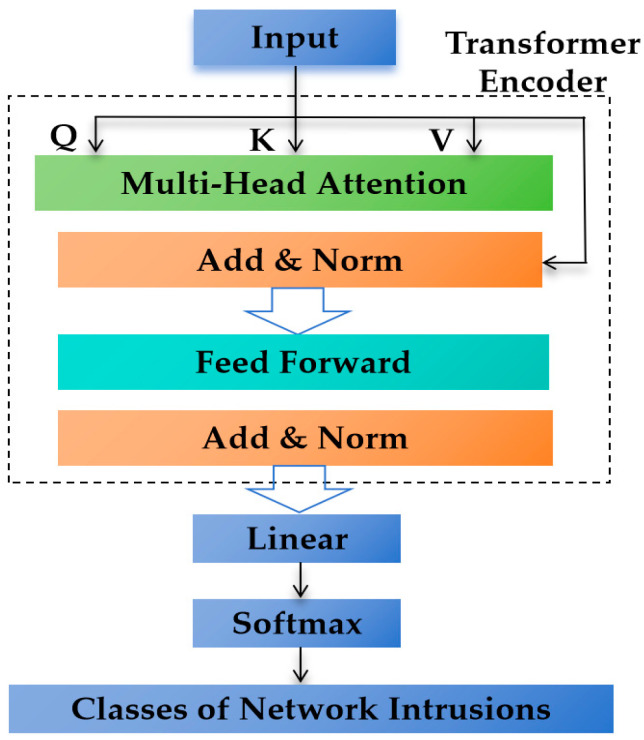
Architecture of a TF-IDM.

The transformer model, renowned for its reliance on the attention mechanism, has achieved considerable success in natural language processing [73]. This architecture is well-suited for network traffic data analysis due to its attention mechanisms that adeptly capture long-range dependencies, allowing the model to focus on salient data segments. A TF-IDM leverages this architecture for network data processing and intrusion detection tasks. As depicted in Figure 2, the canonical transformer structure for an IDS includes an input encoding module for data formatting, multiple transformer blocks for sequential processing, and an output head that condenses the output into a binary classification outcome.

Yin et al. [74] introduced a framework integrating deep capsule networks with attention mechanisms. This approach utilized attention mechanisms to enhance feature extraction quality and the model’s classification performance, resulting in effective outcomes. Liu et al. [75] proposed a capsule network augmented with self-attention from transformers for feature refinement, achieving 97.56% and 95.88% accuracies on CICIDS2017 and NSL-KDD datasets, respectively. Compared to other commonly used traditional intrusion detection models, there is a significant improvement in efficiency. Yao et al. [76] explored a CNN–transformer hybrid for intrusion detection, leveraging transformers for feature relationship learning. Han et al. [77] presented a novel GTID model employing n-gram frequency and a time-aware transformer to extract variable-length session features, demonstrating efficacy on ISCX2012 and CICIDS2017 datasets. Wang et al. [78] combined a ResNet, transformer, and BiLSTM (Res-TranBiLSTM) to detect intrusions by capturing both spatial and temporal traffic characteristics, achieving high accuracies across multiple datasets. Wang and Ullah also harnessed transformers for intrusion detection with promising outcomes. Long et al. [79] developed a transformer-based model to enhance cloud security, attaining over 93% accuracy, which is comparable to CNN-LSTM models, underscoring the transformer’s effectiveness in intrusion detection scenarios.

The TF-IDM employs deep learning’s self-attention mechanism to adeptly handle network traffic data, discerning intricate patterns and enduring dependencies. These models typically exhibit high detection accuracy, particularly in identifying novel and unknown attack patterns. However, they may also encounter challenges, such as high computational resource demands, prolonged model training times, and sensitivity to data imbalances. Nonetheless, with ongoing research and advancements in optimization techniques, transformer-based models demonstrate potential and application value in intrusion detection.

### 4.8. Introduction to BERT-IDMs

BERT [80], short for bidirectional encoder representations from transformers, is a transformative language model predicated on the transformer architecture that was introduced by Google in 2018. It has garnered widespread attention due to its exceptional performance across various natural language processing tasks. In contrast to the GPT model series, BERT utilizes a bidirectional encoder architecture, which means that during training, it takes into account both the text’s preceding and succeeding context, thereby improving its comprehension of contextual relationships. This bidirectional attention mechanism enables BERT to excel in tasks such as question-answering systems, sentence classification, and named entity recognition. Due to its powerful text representation capabilities, BERT has gradually expanded into other domains, including network intrusion detection in cybersecurity.

In a recent study, Nguyen et al. [81] applied the BERT model to the analysis of network traffic sequences to improve the performance of NIDSs. Specifically, the researchers regarded network traffic as sequential data similar to natural language and encoded this data using the BERT model. BERT’s bidirectional attention mechanism captures the interdependencies within traffic sequences, enabling the model to more precisely detect potential threat patterns. The researchers further combined an MLP to classify the features extracted by BERT. This approach effectively differentiates between normal and malicious traffic.

Additionally, the researchers explored the adaptability of the BERT model in cross-domain environments. By training and testing on network traffic data from multiple domains, BERT exhibited robust generalization capabilities, effectively addressing complex attacks in different network environments. To assess its efficacy, the research team conducted experiments on several public datasets, achieving significant results.

Specifically, the BERT-based network intrusion detection method was evaluated on the NSL-KDD and CIC-IDS2017 datasets. The findings indicated that the BERT model attained an accuracy of 97.9% for the NSL-KDD dataset and 95.8% for the CIC-IDS2017 dataset. These results indicated that BERT excels in identifying complex attack patterns, such as malware propagation and data exfiltration, and maintains high detection performance across diverse network environments. Particularly in multi-stage attack detection and cross-domain adaptability, the BERT model has demonstrated its robust generalization capabilities, making it highly effective at managing dynamically changing network threats.

In summary, BERT-based network intrusion detection methods have demonstrated their immense potential in the field of cybersecurity. By leveraging the bidirectional attention mechanism of the BERT model, researchers have not only successfully improved detection accuracy but also significantly enhanced the system’s adaptability and robustness. The experimental results indicate that BERT effectively manages complex network traffic data, excelling in multi-stage attack detection and cross-domain adaptability, thereby highlighting its strengths.

### 4.9. Introduction to GPT-IDMs

GPT-4 [82] and Llama3 [83] are advanced LLMs that have recently emerged, exhibiting remarkable capabilities in natural language processing (NLP). Developed by OpenAI, GPT-4 possesses robust capabilities for generation and comprehension and is capable of handling complex multi-task and multilingual environments. Its training process utilizes large datasets, allowing the model to grasp detailed contextual information and produce high-quality text. Llama3, which was introduced by Meta, is a lightweight LLM that despite having relatively fewer parameters, excels in processing efficiency and adaptability. Through optimized architectural design, Llama3 achieves efficient operation in resource-constrained environments while maintaining high model performance. With their proficiency in handling complex sequential data and producing detailed explanations, these models are progressively being integrated into applications in other domains, such as network intrusion detection in cybersecurity.

A recent investigation by Houssel et al. [84] examined the applicability of GPT-4 and Llama3 for network intrusion detection, leveraging their NLP capabilities to analyze and identify potential threats within network traffic. The researchers regarded network traffic data as sequences akin to natural language and processed them using these models. GPT-4 and Llama3 were able to generate potential threat scenarios and compare them with actual network traffic to detect anomalous behaviors. To enhance detection accuracy, the research team integrated retrieval-augmented generation (RAG) technology. This integration allowed the models to generate detection results and offer detailed explanations and recommendations, helping security analysts better understand the outcomes and take appropriate action. This approach is particularly suitable for complex multi-stage attack detection, achieving high precision in threat identification while maintaining high efficiency.

The researchers conducted experiments utilizing several public network traffic datasets, notably NSL-KDD and CIC-IDS2017. The findings demonstrated that both models excelled in NID, with GPT-4 achieving accuracy rates of 98.2% on the NSL-KDD dataset and 96.7% on CIC-IDS2017, while Llama3 achieved accuracy rates of 97.5% on NSL-KDD and 95.4% on CIC-IDS2017. Llama3 achieved an accuracy rate of 97.5% on the NSL-KDD dataset and 95.4% on the CIC-IDS2017 dataset. These results indicate that these LLMs are highly effective at identifying complex attack patterns (such as DDoS attacks, malware propagation, etc.), with accuracy rates comparable to or higher than traditional machine learning methods. Moreover, the integration of RAG technology greatly improved the interpretability of these models, enabling researchers to produce detailed explanation reports for each detection outcome. This enhancement helps analysts swiftly identify and address potential threats. In multi-stage attack detection tasks, GPT-4 and Llama3 also demonstrated strong generalization capabilities, effectively addressing different threat scenarios in cross-domain environments.

In summary, this study indicates that the application of GPT-4 and Llama3 in network intrusion detection holds tremendous potential. By integrating these advanced language models with NID tasks, researchers have not only improved detection accuracy but also significantly enhanced the system’s interpretability, thereby providing more robust support for cybersecurity analysis. Experimental results on datasets such as NSL-KDD and CIC-IDS2017 demonstrate that these models excel at identifying complex attack patterns and exhibit strong adaptability across various network environments.

### 4.10. Summary

The convergence of DL and intrusion detection has significantly progressed the field of intrusion detection. Unlike traditional machine learning approaches, which require separate stages for feature extraction and classification, DL inherently combines these processes into a single system, thereby eliminating the necessity for manual feature extraction by experts. This integration represents a substantial advantage of DL in this context. Additionally, DL processes large datasets more efficiently and has a higher detection rate and efficiency. Table 2 summarizes the intrusion detection results for different datasets using the nine model methods mentioned above.

## 5. Challenges and Future Trends

### 5.1. Challenges

The development of intrusion detection models in modern network environments mainly faces the following four challenges.

#### 5.1.1. Unavailability of System Datasets

Currently, the majority of proposed methods in the literature are unable to detect zero-day attacks due to insufficient training on a wide range of attack types and patterns. To develop an effective IDS model, it is necessary to test and verify it on datasets with older and newer attacks. Incorporating a comprehensive array of attack definitions into the dataset enables the model to learn diverse patterns, thereby enhancing its capability to protect against various forms of sophisticated intrusions. However, constructing such datasets is costly and resource-intensive, requiring significant expertise. Therefore, a significant challenge in IDS research is systematically creating up-to-date datasets with a comprehensive range of attack instances. Regular updates to the datasets are essential to capture the latest intrusion instances, and such datasets should be made publicly accessible to benefit the research community.

#### 5.1.2. Imbalanced Datasets Leading to Reduced Detection Accuracy

In datasets, low-frequency attacks often have too few samples, which prevents models from adequately learning their characteristics during training, thereby reducing detection accuracy. This also implies that models are ineffective in identifying and classifying new or previously unseen low-frequency attacks. The decline in generalization capability makes models more prone to missing or misjudging low-frequency attacks in practical applications, directly affecting the detection rate. Furthermore, in imbalanced datasets, conventional loss functions (such as cross-entropy loss) fail to effectively address the scarcity of low-frequency samples. Furthermore, the predominance of high-frequency samples often skews the loss function during optimization, leading to an underappreciation of the contributions from low-frequency samples. As a result, the model’s optimization process becomes increasingly biased toward high-frequency samples, while the misclassification loss associated with low-frequency attack samples remains inadequately addressed. This imbalance negatively impacts the detection performance of low-frequency attacks.

#### 5.1.3. Low Performance in Real-World Environments

The research challenges associated with IDSs also encompass their performance in real-world environments. Many proposed methods rely on outdated datasets for laboratory testing and validation, leaving their efficacy in practical scenarios uncertain. It is imperative that once a method has been validated in a controlled setting, it is subsequently evaluated in real-time environments to assess its effectiveness on contemporary networks.

#### 5.1.4. Resources Consumed by Complex Models

Additionally, numerous IDS methods are based on complex models that require substantial processing time and computational resources, leading to increased overhead for processing units and potentially compromising overall IDS performance. While utilizing multicore high-performance GPUs can accelerate computation, this approach incurs higher costs. To mitigate computational overhead, an efficient feature selection algorithm is necessary to identify the most relevant features, thereby optimizing processing speed.

### 5.2. Future Development Trend

There are many areas that warrant further exploration in the future. The challenges mentioned above, such as data imbalances, prolonged packet feature extraction times, and poor interpretability of real data, are all key challenges that need to be addressed in future intrusion detection technologies.

#### 5.2.1. Efficient NIDS Framework

NIDSs are critical defense mechanisms against network intrusions. Recent studies have identified their limitations in detecting zero-day attacks, often resulting in elevated false positive rates. Using the latest systematic and balanced datasets can significantly enhance IDS performance. Exploring this direction is valuable for developing an effective NIDS framework that offers comprehensive intrusion security. The IDS framework should include a mechanism for the regular updating of attack definitions within the dataset, utilizing these updates to continuously train the model and facilitate the learning of new features. This approach will ultimately enhance the model’s capability to detect zero-day attacks while minimizing false positives. Future research directions for NIDS can be explored through two key aspects.

First is the adoption of adversarial training and transfer learning. Over the preceding years, adversarial training has become a prominent approach to enhance model robustness and has steadily attracted attention in the field of intrusion detection. Introducing adversarial examples during the training process can significantly enhance the NIDS’s ability to detect unknown attacks. Future research can further explore how to better integrate adversarial training into intrusion detection models while integrating transfer learning to facilitate model adaptation to novel network contexts and emerging attack vectors.

Second, the development of an adaptive NIDS architecture. As cyber attack patterns and methods continuously evolve, traditional NIDS architectures often struggle to cope with these changes. Therefore, it is crucial to research and design an NIDS architecture with adaptive capabilities. Future research should explore the dynamic adjustment of model parameters based on real-time monitoring of network traffic characteristics to enhance the efficiency and accuracy of detection systems. Such an adaptive architecture has the potential to decrease false positive rates while simultaneously improving the system’s responsiveness to emerging attack vectors.

#### 5.2.2. Updating Datasets and Adapting to the Real Network Environment

Future research will see the generation of more datasets. Chen et al. [32] introduced a new dataset, ZYELL-NCTU NetTraffic-1.0, comprising real, million-level records of daily network traffic rather than being generated from a simulated environment, thereby accurately reflecting authentic network conditions. Although this dataset is still in the learning stage and has not yet achieved optimal performance, it is expected to be utilized for IDS performance evaluation in the future through continuous improvement and training.

Large-scale heterogeneous network datasets typically encompass and include a diverse range of node and edge types, which can more accurately replicate the complexity of real-world scenarios. For instance, Xie et al. [85] have conducted a comprehensive review of the different methods for heterogeneous network representation learning and discussed how to acquire semantic information through various techniques. This is essential for effectively understanding and processing large-scale heterogeneous datasets. In practical applications, data skew is a common issue that may lead to a degradation in model performance. This can be addressed through methods such as federated learning and cross-domain adaptation. Moreover, federated learning and cross-domain adaptation enable models to quickly adapt to new data distributions while maintaining their generalization capabilities for the original tasks.

#### 5.2.3. Optimizing Models in Resource-Constrained Environments

Models utilizing DL algorithms are characteristically complex and require substantial resources, including computing power, storage capacity, and time, which can complicate the deployment of IDSs in real-time scenarios. One approach to mitigating these challenges is the use of high-performance GPUs for the rapid and efficient processing of large datasets, although the high cost of these GPUs presents a performance–cost trade-off. To manage expenses, exploring cloud-based GPU services for model training may be advantageous. Additionally, reducing the complexity of deep learning models through effective and intelligent feature engineering could further drive advancements in the field of network intrusion detection. Ultimately, this will simplify the model and reduce its computing resource requirements in real-time environments. Furthermore, the integration of quantum computing and edge computing, along with the adoption of lightweight model architectures and model optimization, can drive development within the realm of network intrusion detection in forthcoming research.

In existing solutions, techniques such as model pruning, knowledge distillation, and lightweight architectures (e.g., MobileNet and EfficientNet) can significantly reduce the number of model parameters and computational demands, all while preserving detection accuracy. Technologies like pruning and quantization are employed to decrease the model’s parameter count and computational load. For instance, network pruning simplifies model complexity by removing redundant neurons or connections, while quantization techniques convert floating-point weights into low-precision integers, thereby lowering the model’s storage and computational requirements. Lightweight network architectures, such as MobileNet, SqueezeNet, and TinyML, are specifically designed for resource-constrained devices. They optimize the network structure to significantly reduce the model size and computational cost while maintaining accuracy. Knowledge distillation (KD) facilitates model size reduction by training a smaller “student” model to replicate the outputs of a larger “teacher” model, thereby minimizing any adverse effects on performance. For example, Wang et al. [86] introduced a BERT-TPF-based knowledge distillation model aimed at intrusion detection in the IoT. This methodology yields a compact final model requiring only 788 parameters, marking a reduction of approximately 90% compared to earlier models. Remarkably, this model attained accuracy rates exceeding 99% on both the CIC-IDS2017 and TON_IoT datasets.

Despite the existing technologies having addressed the issue of model size reduction to some extent, challenges still persist in practical applications. Future research could further explore the following directions.

First, dynamic model adjustment facilitates the real-time modification of model complexity based on the operational status of IoT devices, such as battery levels and computational loads [87]. This capability allows for a more responsive approach to optimizing the balance between performance and energy efficiency, ensuring that the models can adapt to varying conditions without sacrificing their effectiveness. For instance, when an IoT device is operating under a low battery condition, the model can automatically reduce its complexity to conserve energy while still maintaining adequate performance levels. This adaptability is crucial in IoT environments in which devices often have limited resources and need to operate efficiently over extended periods.

Second, there is the concept of cross-domain optimization [88]. This approach makes it feasible to develop universal model optimization methods that can be applied across various types of IoT devices. By creating models that are versatile enough to operate efficiently on different hardware platforms, researchers can streamline the deployment process and reduce the time and effort required to customize models for specific devices. This universality not only enhances the scalability of deep learning applications in IoT but also promotes interoperability among diverse devices and systems.

Additionally, as mentioned earlier, hardware acceleration plays a significant role in enhancing model performance [89]. By integrating hardware optimizations, such as utilizing low-power chips specifically designed for deep learning—like the FPGA, Edge TPU, and NVIDIA Jetson Nano—the execution efficiency of models on IoT devices can be significantly improved. These specialized hardware solutions enable faster processing times and reduced energy consumption, which are essential for maintaining the overall effectiveness of NIDSs in real-time scenarios.

As quantum computing continues to advance, its potential benefits for handling complex computational tasks are becoming increasingly evident [90]. Future research can delve into how quantum computing can accelerate the training process of deep learning models, particularly in the context of large-scale datasets that are common in IoT environments. The unique capabilities of quantum computing may allow for more efficient algorithms that can process vast amounts of data more quickly than traditional methods.

Moreover, edge computing, as a distributed computing paradigm, can significantly reduce data transmission latency and bandwidth usage. By deploying lightweight NIDS models at the network edge, organizations can enhance the system’s real-time detection capabilities and response speed. This localized processing minimizes the need to send large volumes of data to centralized servers, thereby improving efficiency and reducing the risk of data breaches during transmission. Future research can also focus on optimizing the complexity of NIDS models to better adapt to resource-constrained practical application scenarios. The integration of these advanced technologies will provide more practical solutions for implementing NIDSs in embedded devices or IoT environments, ultimately leading to more secure and efficient systems.

#### 5.2.4. Evolution and Integration of NIDSs

There has been a notable increase in scholarly interest in information physical systems (CPSs), particularly supervisory control and data acquisition systems (SCADAs) and unmanned aerial vehicle (UAV) networks. SCADA systems are widely employed in applications such as smart grid management. However, the integration of advanced information and communication technologies has led to increased complexity within SCADA networks, thereby creating new vulnerabilities that can be exploited by attackers. NIDSs are vital in these environments, as they enable the detection of intruders through network traffic analysis. The application of ML and DL can significantly enhance NIDS efficiency by introducing additional analytical dimensions for detecting network attacks within SCADA networks. Nonetheless, this domain is still evolving, and further research is necessary to design and implement effective ML- and DL-based NIDSs tailored for SCADA environments. Given the inherent characteristics of communication in wireless channels, these networks are accessible to both authorized users and potential intruders, who can monitor communications and initiate various forms of attacks. Thus, the development of robust and intelligent NIDSs is critical for the detection of intruders in UAV networks. Additionally, examining the application of AI in NIDSs for UAV networks represents a promising avenue for research.

In practical terms, NIDSs can be employed across various contexts, including the Industrial Internet of Things (IIoT) [91,92] and smart cities [93,94]. As the IIoT continues to evolve, a growing number of industrial devices are being interconnected, which poses new cybersecurity challenges. Future research should focus on creating effective NIDS frameworks that address the specific requirements of the IIoT environment, ensuring the security of industrial control systems. Furthermore, the integration of deep learning and big data analytics can facilitate real-time monitoring and anomaly detection of the extensive data generated by these devices, thus enhancing overall system security and stability. Smart cities, by leveraging IoT technology, connect multiple systems such as transportation, energy management, and public safety, which also exposes urban networks to greater security threats. Future research in this domain could focus on creating NIDS systems designed for large-scale, heterogeneous network environments and integrate AI technologies to implement intelligent intrusion detection and response mechanisms.

Overall, research directions in network intrusion detection using DL encompass model enhancement and innovation, data fusion and processing, adversarial defense, and zero-day vulnerability detection. These studies aim to enhance the efficiency and robustness of network security protection systems.

## 6. Conclusions

Through the study of intrusion detection technologies and related models, DL techniques have provided more advanced solutions compared to traditional intrusion detection methods. This paper offers a summary and analysis of the recent research landscape regarding IDSs that employ DL, addressing key areas such as intrusion detection datasets, data preprocessing techniques, and model classification. Although numerous innovative and effective methods have been proposed and implemented, there remains potential for enhancing detection performance in practical applications. Given the rapid advancement of IoT devices and complex network environments, future deep learning-based intrusion detection models will need to not only efficiently, rapidly, and accurately identify complex network traffic but also address practical challenges, such as reducing model size and optimizing performance in resource-constrained environments. Therefore, future research will focus on optimizing computational resources and response times of the models while maintaining high detection accuracy to better adapt to evolving network threats and application scenarios.

## Figures and Tables

**Table 1:** Summary of seven common datasets.

Dataset	Year of Creation	Numbers of Network Attacks	Attack Types
KDDCUP99	1998	4	Probe, DOS, U2R, R2L
NSL-KDD	2009	4	DoS, Probe, R2L, U2R
UNSW-NB15	2015	9	Backdoors, DoS, Exploits, Fuzzers, Generic, Analysis, Reconnaissance, Shellcode, Worms
CIC-IDS2017	2017	7	Brute Force, HeartBleed, Botnet, DoS, DDoS, Web, Infiltration
Kyoto2006+	2006	3	Normal, Attacks, Unknown Attacks
ISCX2012	2012	4	HTTPDos, DDos using an IRC botnet, SSH brute force
MQTTet2020	2020	5	DoS, Brute force, Malformed, SlowITe, Flood

**Table 2:** Representative works of intrusion detection methods based on DL.

Refs	DL Technology	Feature Election/Extraction	Dataset	Task Classes	Performance Evaluation
[33]	AE	AE	KDDCup99	BC;MC	BC: ACC: 96.53%, FPR: 0.35% MC: 94.71%
[38]	DAE	DAE	NSL-KDD; KDDCup99	MC	NSL-KDD: ACC: 85.42%, Recall: 85.42%, F1: 87.37% KDDCup99: ACC: 97.85%, Recall: 97.85%, F1: 98.15%
[39]	SDAE	SDAE	KDDCup99; UNSW-NB15	MC	KDDCup99: ACC: 99.996%, FPR: 0.00001% UNSW-NB15: ACC: 89.134%, FPR: 0.7495%
[40]	SSAE	SSAE	NSL-KDD	MC	ACC: 99.35%, Recall: 99.01%, FPR: 0.13% Recall: 99.43% (Normal), 99.35% (Dos), 99.03% (Probe), 83.43% (R2L), 67.94% (U2R)
[46]	DBN	DBN	10%KDDCup99	BC	ACC: 95.45%
[47]	DBN	DBN	CIC-IDS2017	MC	Precision: 97.8%, Recall: 97.73%, F1: 97.74%
[48]	DBN, PNN	DBN	KDDCup99	MC	ACC: 99.14%, DR: 93.25%, FPR: 0.615%
[49]	DBN	DBN	NSL-KDD; UNSW-NB15	MC	NSL-KDD: ACC: 82.08%, Recall: 70.51%, Precision: 97.27%, F1: 81.75%, FPR: 2.62% UNSW-NB15: ACC: 90.21%, Recall: 90.22%, Precision: 87.3%, F1: 91.54%, FPR: 17.15%
[50]	DBN	GA	NSL-KDD	MC	ACC: 98.82%, Recall: 97.67%, FAR: 2.65%
[51]	DBN, KELM	DBN	KDDCup99; NSL-KDD;UNSW-NB15;CIC-IDS2017	BC;MC	BC: KDDCup99: Precision: 94%, Recall: 98.73%, F1: 96.31% NSL-KDD: Precision: 93.64%, Recall: 98.4%, F1: 96.6% UNSW-NB15: Precision: 82.3%, Recall: 96.4%, F1: 88.79% CIC-IDS2017: Precision: 96.8%, Recall: 98.19%, F1: 97.49%
[53]	DNN	SC	KDDCup99;NSL-KDD	MC	ACC: 92.1%, Recall: 92.23%
[54]	DNN	SMO	KDDCup99; NSL-KDD	BC	NSL-KDD: Precision: 99.5%, Recall: 99.5%, F1: 99.6% KDDCup99: Precision: 92.7%, Recall: 92.8%, F1: 92.7%
[55]	AE, DNN	ICVAE	NSL-KDD; UNSW-NB15	MC	UNSW-NB15: ACC: 89.08%, Precision: 86.05%, Recall: 95.68%, F1: 90.61%, FPR: 19.01% NSL-KDD: ACC: 85.97%, Precision: 97.39%, Recall: 77.43%, F1: 86.27%, FPR: 2.74%
[56]	CNN	CNN	KDDCup99	BC	ACC: 99.23%
[57]	CNN	CRF, LCFS	KDDCup99	MC	Precision: 98.88%
[58]	CNN	CNN	NSL-KDD	MC	ACC: 70.09%, FPR: 2.35% (Dos), 2.09% (Probe), 0.69% (R2L), 0.06% (U2R), Recall: 83.21% (Dos), 81.87% (Probe), 21.68% (R2L), 13% (U2R)
[59]	CNN	CNN	UNSW-NB15; CIC-IDS2017	BC;MC	BC: UNSW-NB15: Recall: 99.74% MC: UNSW-NB15: Recall: 96.54% CIC-IDS2017: Recall: 99.85%
[60]	CNN	CNN	NSL-KDD; CIC-IDS2017	MC	NSL-KDD: ACC: 96.1%, Recall: 92.3%, FPR: 2%, F1: 94% CIC-IDS2017: ACC: 95.6%, Recall: 94.1%, FPR: 5%, F1: 94.6%
[61]	CNN, LSTM	\	ISCX2012	MC	ACC: 99.69%, Recall: 96.91%, FPR: 0.22%
[64]	LSTM	\	NSL-KDD	MC	ACC: 84.25%, Recall: 97.5%, FPR: 25.7%
[65]	LSTM, AE	AE	ISCX2012	BC	F1: 85.83%
[66]	GRU	\	Kyoto2006+	BC	ACC: 84.15%
[67]	GRU, MLP	GRU	NSL-KDD; KDDCup99	MC	NSL-KDD: ACC: 99.24%, Recall: 99.31%, FPR: 0.84% KDDCup99: ACC: 99.84%, Recall: 99.42%, FPR: 0.05%
[68]	CNN, RNN, LSTM, GRU	CNN	NSL-KDD	MC	CNN-2layer-LSTM: ACC: 99.7%, Precision: 99.9%, Recall: 99.6%, F1: 99.8% CNN-2layer-GRU: ACC: 98.1%, Precision: 99.9%, Recall: 97.6%, F1: 98.8% CNN-2layer-RNN: ACC: 97.3%, Precision: 100%, Recall: 96.7%, F1: 98.3%
[69]	CNN, LSTM	CNN	KDDCup99	MC	ACC: 86.4%
[71]	DBSCAN + GAN	PCA	NSL-KDD; UNSW-NB15;Kyoto2006	MC	ACC: 98.65%
[75]	Transformer	Self-attention	NSL-KDD;CIC-IDS2017	MC	NSL-KDD: ACC: 95.88%, F1: 95.87% CIC-IDS2017: ACC: 97.56%, F1: 97.74%
[77]	Transformer	DNN, Transformer	ISCX2012;CICIDS2017	MC	ISCX2012: Accuracy: 99.42, Precision: 99.41, Recall99.34, F1: 99.37 CICIDS2017: Accuracy: 97.87, Precision: 98.16, Recall: 97.59, F1: 97.83
[81]	BERT	\	NSL-KDD;CIC-IDS2017	MC	NSL-KDD: Accuracy: 97.9% CIC-IDS2017: Accuracy: 95.8%
[84]	GPT-4, Llama3	\	NSL-KDD;CIC-IDS2017	MC	GPT-4: NSL-KDD: Accuracy: 98.2%;CIC-IDS2017: Accuracy: 96.7% Llama3: NSL-KDD: Accuracy: 97.5%;CIC-IDS2017: Accuracy: 95.4%

notes: binary classification (BC); multi-classification (MC), “\”indicates that this method did not participate in the evaluation.

## Data Availability

The data presented in this study are contained in the article itself.
